# Mitochondrial Impairment May Increase Cellular NAD(P)H: Resazurin Oxidoreductase Activity, Perturbing the NAD(P)H-Based Viability Assays

**DOI:** 10.3390/cells4030427

**Published:** 2015-08-21

**Authors:** Vasily A. Aleshin, Artem V. Artiukhov, Henry Oppermann, Alexey V. Kazantsev, Nikolay V. Lukashev, Victoria I. Bunik

**Affiliations:** 1 Faculty of Bioengineering and Bioinformatics, Lomonosov Moscow State University, Moscow 119234, Russia; E-Mails: aleshin_vasily@mail.ru (V.A.A.); whitelord32br@gmail.com (A.V.A.); 2 Neurosurgical clinic and polyclinic, Leipzig University Clinic, Leipzig 04103, Germany; E-Mail: henry.oppermann@medizin.uni-leipzig.de; 3 Faculty of Chemistry, Lomonosov Moscow State University, Moscow 119234, Russia; E-Mails: mak@org.chem.msu.ru (A.V.K.); nvlukashev@gmail.com (N.V.L.); 4 A.N. Belozersky Institute of Physicochemical Biology, Lomonosov Moscow State University, Moscow 119234, Russia

**Keywords:** glioblastoma viability, cellular NAD(P)H-dependent oxidoreductase, metabolon, thiamin, oxythiamin, 2-oxo acid dehydrogenase, phosphonate analog of 2-oxo acid, resazurin, T98G, U87

## Abstract

Cellular NAD(P)H-dependent oxidoreductase activity with artificial dyes (NAD(P)H-OR) is an indicator of viability, as the cellular redox state is important for biosynthesis and antioxidant defense. However, high NAD(P)H due to impaired mitochondrial oxidation, known as reductive stress, should increase NAD(P)H-OR yet perturb viability. To better understand this complex behavior, we assayed NAD(P)H-OR with resazurin (Alamar Blue) in glioblastoma cell lines U87 and T98G, treated with inhibitors of central metabolism, oxythiamin, and phosphonate analogs of 2-oxo acids. Targeting the thiamin diphosphate (ThDP)-dependent enzymes, the inhibitors are known to decrease the NAD(P)H production in the pentose phosphate shuttle and/or upon mitochondrial oxidation of 2-oxo acids. Nevertheless, the inhibitors elevated NAD(P)H-OR with resazurin in a time- and concentration-dependent manner, suggesting impaired NAD(P)H oxidation rather than increased viability. In particular, inhibition of the ThDP-dependent enzymes affects metabolism of malate, which mediates mitochondrial oxidation of cytosolic NAD(P)H. We showed that oxythiamin not only inhibited mitochondrial 2-oxo acid dehydrogenases, but also induced cell-specific changes in glutamate and malate dehydrogenases and/or malic enzyme. As a result, inhibition of the 2-oxo acid dehydrogenases compromises mitochondrial metabolism, with the dysregulated electron fluxes leading to increases in cellular NAD(P)H-OR. Perturbed mitochondrial oxidation of NAD(P)H may thus complicate the NAD(P)H-based viability assay.

## 1. Introduction

Pyridine nucleotides, such as nicotinamide adenine dinucleotide or its ribose-2′-phosphorylated derivative, are universal electron donors/acceptors in cellular processes. They undergo reversible oxidation-reduction in a number of biological reactions and are therefore abundant in cells. The majority of cellular dehydrogenases use either the oxidized, NAD(P)^+^, or reduced, NAD(P)H, form of pyridine nucleotides as a substrate. Electrons from catabolic pathways are usually transferred to NAD^+^, while NADPH is used for cellular biosynthesis, with the electron exchange between NADH and NADPH catalyzed by transhydrogenases. The ratio of NAD(P)H/NAD(P)^+^ is tightly linked to the less abundant and/or more cell-specific redox components of a cell, such as FADH_2_/FAD or lactate/pyruvate couples. As a result, the steady-state ratio NAD(P)H/NAD(P)^+^ is an important parameter of cellular physiology, integrating information on the catabolic efficiency and biosynthetic potential of a cell. It is therefore not surprising that natural homeostatic mechanisms employ pyridine nucleotides in signaling reactions. They include formation of secondary messengers, such as nicotinic acid adenine dinucleotide phosphate (NAADP^+^) [1] or ROS [2,3], and regulation of protein post-translational modifications, such as deacetylation of proteins by sirtuins [4,5,6], or mono- and poly-ADP-ribosylation of proteins upon DNA damage [7]. While redox reactions change the NAD(P)H/NAD(P)^+^ ratio reversibly, the signaling processes mentioned above also affect the total pyridine nucleotide pool by consuming NAD^+^. As a result, dysfunctional cells are characterized by a changed level of reduction and depletion of total pool of the pyridine nucleotides, which prime the cells to enter the death pathway.

This phenomenon underlies existing methods of monitoring cellular viability through the NAD(P)H-dependent reduction of artificial dyes—a side reaction catalyzed by a number of cellular dehydrogenases [8,9]. A decreased flux of electrons to this side reaction may be due to both the lower level of NAD(P)H in each cell of low viability and the associated reduction in total cell number. Hence, the side reaction may be employed to measure cellular viability and death. However, the interpretation of an increased flux of electrons to the dye reduction is less obvious and usually not considered. Is the increase a sign of a higher biosynthetic and antioxidant potency, or of a disturbed homeostasis, such as reductive stress? While oxidative stress is characterized by insufficient NAD(P)H due to increased ROS production, reductive stress refers to increased NAD(P)H which cannot be properly oxidized. Reductive stress also leads to increased ROS, reflecting cellular inability to use the high redox for normal oxidative metabolism. This is known to occur under pathological conditions, such as hypoxia or dysfunction of the NADH-oxidizing mitochondrial enzymes, e.g., complex I and downstream components of the respiratory chain. However, perturbation of NAD(P)H oxidation due to organization of metabolic pathways within supramolecular enzyme structures, metabolons [10,11,12], has received less attention in this regard.

We hypothesize that structural organization of metabolic pathways causes non-equivalence of the NAD(P)H sources for the NAD(P)H oxidation pathways, such that oxidizing enzymes preferably use NAD(P)H from their partner producers. Hence, if a partner NAD(P)H producer is inactivated, the evolutionary optimized metabolic flux cannot be re-established *in vivo* due to the chemistry-driven increase of the NAD(P)H production from other sources. The sub-optimal oxidation of NAD(P)H outside specific metabolons may therefore lead to reductive stress also when the NAD(P)H producers are inhibited, while the NAD(P)H oxidizers are not. In the present work, we test this hypothesis using a model of metabolic impairment caused by inhibition of the NAD(P)H producers. Cells were treated with inhibitors of the mitochondrial NADH-producing 2-oxo acid dehydrogenases or with oxythiamin, which inhibits not only the 2-oxo acid dehydrogenases, but also transketolase essential for cytosolic NADPH production in the pentose phosphate shuttle. Applying the inhibitors, we could observe the condition-dependent increases of the electron flux to a tetrazolium dye resazurin (Alamar Blue). Cellular reduction of the dye to resorufin, catalyzed by intracellular NAD(P)H-dependent oxidoreductases, is used to test cellular viability in commercially available tests, such as the CellTiterBlue test (Promega) used in our work. Our data point to the significance of the intact mitochondrial metabolism and metabolic interaction between mitochondria and cytosol for the resazurin reduction to be a measure of cellular viability. When the NADH production in the tricarboxylic acid cycle and affiliated 2-oxo acid dehydrogenase reactions is disturbed, other reactions can compensate for the NAD(P)H normally produced by these enzymes. As a result, the resazurin reduction by cells is constant or even increased, but this does not correspond to unchanged or higher cellular viability. Rather, the electron flux to the dye may increase due to perturbed mitochondrial network of the NAD(P)H-dependent reactions. Appropriate caution is thus required when using resazurin reduction as a measure of cellular viability.

## 2. Experimental Section

### 2.1. Synthesis of the Phosphonate Analogs of Pyruvate

*O*,*O*′-Dimethyl acetylphosphonate (**AcPMe_2_**) was obtained according to [13]. A mixture of dimethyl phosphite and ethyl vinyl ether was added dropwise to acetyl chloride at 0 °C. The reaction mixture was stirred at ambient temperature for 48 h. The product was purified by vacuum distillation with 72% yield, b.p. 47–48 °C/0.9 mm, purity >95%. ^1^H-NMR (400 MHz, CDCl_3_), δ, ppm: 3.84 (d, *J* = 10.8 Hz, 6H, (CH3O)_2_P(O)), 2.46 (d, *J* = 5.3 Hz, 3H, C(O)CH_3_); ^31^P-NMR (161.9 MHz, CDCl_3_), δ, ppm: −1.0.

*O*-Methyl sodium acetylphosphonate (**AcPMe**) was prepared according to [14]. Sodium iodide in dry acetone was added dropwise to a solution of *O*,*O*′-dimethyl acetylphosphonate in dry acetone with stirring at 0 °C. The reaction mixture was stirred for 18 h at ambient temperature. The precipitate was filtered off, washed with dry acetone and dried under vacuum. The yield of *O*-methyl sodium acetylphosphonate was 94%, m.p. 190–191 °C, purity >97%. ^1^H-NMR (400 MHz, DMSO-*d*_6_), δ, ppm: 3.34 (d, *J* 10.0 Hz, 3H, (CH_3_O)P(O)), 2.15 (d, *J* 3.5 Hz, 3H, C(O)CH_3_); ^13^C-NMR (100.6 MHz, D_2_O), δ, ppm: 220.1 (d, *J* 163.6 Hz, C(O)CH_3_), 52.9 (d, *J* 5.9 Hz, (CH_3_O)P(O)), 30.3 (d, *J* 49.7 Hz, C(O)CH_3_); ^31^P-NMR (161.9 MHz, DMSO-*d_6_*), δ, ppm: −0.5.

### 2.2. Synthesis of the Phosphonate Analogs of 3-Methyl-2-Oxovalerate (“Ketoisoleucine”)

*O*-Methyl sodium (2-methylbutanoyl)phosphonate (**2-MBPMe**) was prepared from *O*,*O*′-dimethyl (2-methylbutanoyl)phosphonate and sodium iodide in dry acetone according to [14] with 74% yield, m.p. 205–206 °C, and purity >97%. ^1^H-NMR (400 MHz, D_2_O), δ, ppm: 3.65 (d, *J* = 10.5 Hz, 3H, (CH_3_O)P(O), 3.14 (m, 1H, CHCH_3_), 1.79 (m, 1H, CH_2_CH_3_), 1.49 (m, 1H, CH_2_CH_3_), 1.13 (d, *J* = 7.0 Hz, 3H, CHCH_3_,), 0.91 (t, *J* = 7.5 Hz, 3H, CH_2_CH_3_); ^13^C-NMR (100.6 MHz, D_2_O), δ, ppm: 226.0 (d, *J* = 154.3 Hz, C(O)CH), 52.9 (d, *J* = 5.9 Hz, (CH_3_O)P(O)), 47.5 (d, *J* = 43.8 Hz, CHCH_3_), 24.7 (CH_2_CH_3_), 14.4 (CH(CH_3_)), 10.9 (CH_2_CH_3_); ^31^P-NMR (161.9 MHz, D_2_O), δ, ppm: −0.1. The precursor *O*,*O*′-dimethyl (2-methylbutanoyl)phosphonate was prepared according to [13] from dimethyl phosphite, ethyl vinyl ether, and 2-methylbutanoyl chloride with 60% yield, b.p. 60–61 °C/0.9 mm. ^1^H-NMR (400 MHz, CDCl_3_), δ, ppm: 3.84 (d, *J* = 10.7 Hz, 6H, (CH_3_O)_2_P(O)), 3.01 (m, 1H, CHCH_3_), 1.83 (m, 1H, CH_2_CH_3_), 1.44 (m, 1H, CH_2_CH_3_), 1.11 (d, *J* = 7.0 Hz, 3H, CHCH_3_,), 0.89 (t, *J* = 7.5 Hz, 3H, CH_2_CH_3_,); ^13^C-NMR (100.6 MHz, CDCl_3_), δ, ppm: 213.9 (d, *J* = 155.9 Hz, C(O)CH), 53.8 (d, *J* = 6.7 Hz, (CH_3_O)P(O)), 53.7 (d, *J* = 6.7 Hz, (CH_3_O)P(O)), 48.1 (d, *J* = 52.3 Hz, CHCH_3_), 24.4 (CH_2_CH_3_), 14.2 (CH(CH_3_)), 11.2 (CH_2_CH_3_); ^31^P-NMR (161.9 MHz, CDCl_3_), δ, ppm: −0.9.

*O*,*O*′-Diethyl (2-methylbutanoyl)phosphonate (**2-MBPEt_2_**) was synthesized from triethyl phosphite and 2-methylbutyryl chloride. The reaction mixture was stirred at ambient temperature for 18 h. The product was isolated by vacuum distillation with 90% yield, b.p. 69–72 °C/0.9 mm, and purity >97%. ^1^H-NMR (400 MHz, CDCl_3_), δ, ppm: 4.20 (m, 4H, (CH_3_CH_2_O)_2_P(O)), 3.04 (m, 1H, CHCH_3_), 1.83 (m, 1H, CH_2_CH_3_), 1.44 (m, 1H, CH_2_CH_3_), 1.35 (t, *J* = 7.0 Hz, 6H, (CH_3_CH_2_O)_2_P(O)), 1.13 (d, *J* = 7.0 Hz, 3H, CHCH_3_,), 0.89 (t, *J* = 7.5 Hz, 3H, CH_2_CH_3_,); ^13^C-NMR (100.6 MHz, CDCl_3_), δ, ppm: 214.6 (d, *J* = 156.8 Hz, C(O)CH), 63.5 (d, *J* = 5.1 Hz, (CH_3_CH_2_O)P(O)), 63.4 (d, *J* = 5.1 Hz, (CH_3_CH_2_O)P(O)), 47.9 (d, *J* = 53.1 Hz, CHCH_3_), 24.5 (CH_2_CH_3_), 16.3 (d, *J* = 5.9 Hz, (CH_3_CH_2_O)_2_P(O)), 14.5 (CH(CH_3_)), 11.3 (CH_2_CH_3_); ^31^P-NMR (161.9 MHz, CDCl_3_), δ, ppm: −2.8.

### 2.3. Cellular NAD(P)H:Resazurin Oxidoreductase Assay

Human glioblastoma cell lines T98G and U87 were obtained from the American Type Culture collection (LGC Standards GmbH; Wesel, Germany). Cells at a density of 2.5 × 10^4^ cells/mL, 200 μL per well, were seeded on black microplates with clear bottom (Greiner, μClear^®^, Frickenhausen, Germany) in DMEM (4.5 g/L glucose, supplemented with 10% FCS, 2 mM GlutaMAX™ (Invitrogen, Carlsbad, CA, USA) and antibiotics). After 24 h, the medium was exchanged for 100 μL per well of Hank’s buffered salt solution, HBSS (1 g/L glucose, 0.37 M NaCl, 5.4 mM KCl, 0.25 mM Na_2_HPO_4_, 0.44 mM KH_2_PO_4_, 1.3 mM CaCl_2_, 1.0 mM MgSO_4_, 4.2 mM NaHCO_3_), or an equal amount of DMEM (1 g/L glucose, supplemented with 2 mM GlutaMAX™). Phosphonate analogs, thiamin or oxythiamin were added at different concentrations (0.01–20 mM) into the media specified in the figure legends. After incubation for 5 h or 24 h, the NAD(P)H:resazurin oxidoreductase activity was determined in the control and treated wells using the CellTiterBlue assay according to manufacturer’s recommendations. The CellTiterBlue reagent was added in a freshly exchanged medium omitting inhibitors, and the dye reduction was allowed to proceed for 90 min at 37 °C. In each experiment, fluorescence from four wells was averaged and NAD(P)H:resazurin oxidoreductase rates of the treated cells were expressed as percentage of the rates exhibited by the control cells. Effects of the phosphonate analogs of 2-oxo acids were screened using five independent cell batches for each culture, with specific concentration dependences repeated up to three times. The thiamin and oxythiamin treatments were performed in ≥4 independent experiments.

### 2.4. Enzyme Assays in Cell Lysates

Cells were seeded on tissue culture dishes (TPP) at a density of 10^5^ cells/mL, 10 mL per dish, in DMEM (4.5 g/L glucose, supplemented with 10% FCS, 2 mM GlutaMAX™ and antibiotics). After 24 h cell medium was exchanged for an equal amount of medium with thiamin or oxythiamin (0.05 or 5 mM). 5 h or 24 h later cell medium was substituted for 200 μL of lysis buffer, comprising 50 mM Tris-HCl pH 7.5, 150 mM NaCl, 1% IGEPAL CA-630 (Sigma, St Louis, MO, USA), and the protease and phosphatase inhibitor cocktails (cOmplete and PhosSTOP, Roche, Basel, Switzerland). Cells were scrapped, and the suspensions collected into 1.5 mL eppendorf tubes. The cell lysates were used for the enzyme activity measurements after incubation on ice for at least 10 min.

The 2-oxoglutarate dehydrogenase complex (OGDH), NADP^+^-dependent malic enzyme, glutamate dehydrogenase (GDH) and malate dehydrogenase (MDH) were tested spectrophotometrically at 340 nm by the NAD(P)H production/consumption rate on the day of the lysate preparation. OGDH was assayed in 20 mM potassium-phosphate buffer, pH 7.0, containing 1 mM ThDP, 1 mM MgCl_2_, 2.5 mM NAD^+^, 1 mM dithiothreitol, 1 mM CaCl_2_, 0.1 mM CoA and 2 mM 2-oxoglutarate. GDH was assayed in 100 mM Tris-HCl, pH 7.5, containing 0.2 mM NADH, 50 mM NH_4_Cl and 2.5 mM 2-oxoglutarate. MDH was assayed in 20 mM potassium-phosphate buffer, pH 7.2, containing 0.14 mM NADH and 0.3 mM oxaloacetate. NADP^+^-dependent malic enzyme was assayed in 100 mM Tris-HCl, pH 7.5, containing 0.4 mM NADP^+^, 4mM MgCl_2_, and 5 mM malate. The linear part of the product accumulation curves was used for the reaction rate determination. The linearity held during the first 2–4 min (MDH) or 20–30 min (OGDH, GDH, malic enzyme) of the reaction. The lysates were assayed at two different protein concentrations, each concentration in quadruplicate. The enzyme aliquot added to the medium omitting the acid substrate was used as a blank. Pyruvate dehydrogenase (PDH) activity was tested using Pyruvate dehydrogenase Protein Quantity Dipstick Assay Kit (Abcam Inc., Cambridge, MA, USA) according to manufacturer’s recommendations, using the lysates stored at −20 °C.

### 2.5. Protein Assay in Cell Lysates

Protein concentration in cell lysates was measured in triplicate using Pierce™ (Life Technologies, Gaithersburg, MD, USA) 660 nm Protein Assay and bovine serum albumin (Sigma) as a standard.

### 2.6. Statistical Analysis

Significant differences with control upon multiple group comparison were determined at *p* < 0.05 by one-way ANOVA followed by *post-hoc* analysis using Dunnett’s test for the data on concentration dependencies, or Tuckey’s test in other cases. Student’s *t*-test was used to compare two groups. The analysis was done with GraphPad Prism version 5.00, GraphPad Software, Inc (San Diego, CA, USA). Non-linear regression of the concentration dependencies was made using SigmaPlot 12.0, Systat Software (Point Richmond, CA, USA).

## 3. Results and Discussion

### 3.1. Influence of Thiamin and Its Antagonist Oxythiamin on Cellular NAD(P)H:Resazurin Oxidoreductase

**Figure 1:**
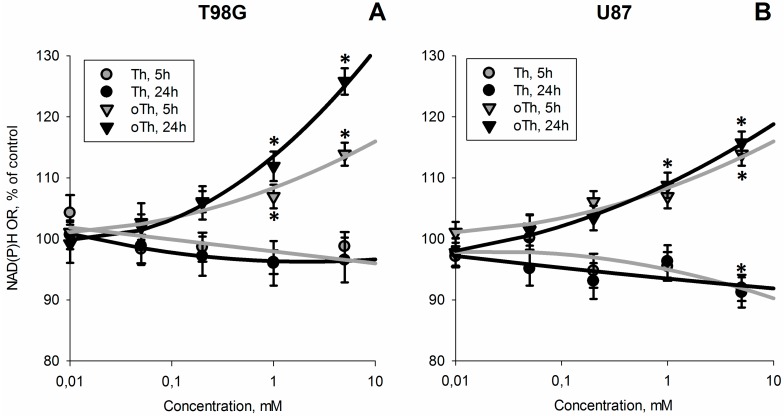
Concentration- and time-dependent changes of NAD(P)H:resazurin oxidoreductase activity (NAD(P)H OR) of T98G (**A**) and U87 (**B**) cells incubated with different concentrations of thiamin (Th) or oxythiamin (oTh). Incubation time (5 h or 24 h) is indicated on the graphs. Changes relative to the control values are presented as mean ± SEM, %. Significant differences with control (* *p* < 0.05) were determined by one-way ANOVA followed by *post-hoc* analysis using Dunnett’s Multiple Comparison Test.

**Figure 2:**
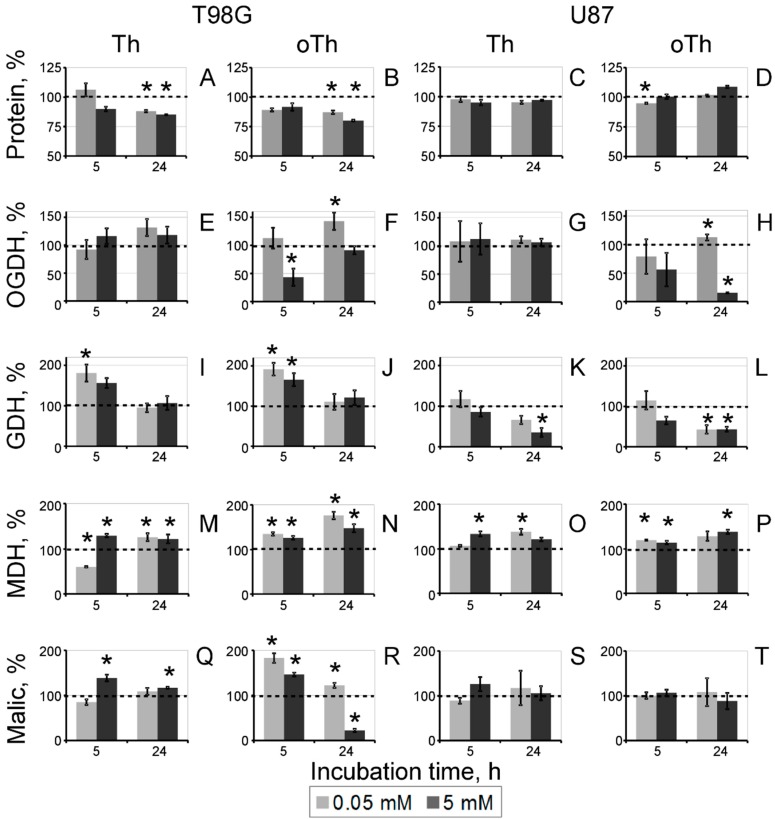
Concentration- and time-dependent changes in cellular protein (**A**–**D**) and activities of the ThDP-dependent (OGDH, **E**–**H**) and affiliated (GDH, **I**–**L**; MDH, **M**–**P**; malic, **Q**–**T**) enzymes upon incubation of T98G (left panel) and U87 (right panel) cells with thiamin (Th) and oxythiamin (oTh). Changes relative to the control values (Table 1) are shown as mean ± SEM, %. Significant differences with control (* *p* < 0.05) in the data groups at a fixed incubation time (5 h or 24 h) were determined by one-way ANOVA followed by *post-hoc* analysis using Dunnett’s Multiple Comparison Test.

Figure 1 shows that incubation of cells with the thiamin antagonist oxythiamin increases cellular NAD(P)H:resazurin oxidoreductase, while similar concentrations of thiamin do not cause this effect. As shown in Figure 2, both thiamin and oxythiamin slightly (up to 20%) decrease cellular protein in T98G cells, which is not expressed in U87 cells. Hence, the opposite action of thiamin and oxythiamin on cellular NAD(P)H:resazurin oxidoreductase in both cell lines, cannot be due to the changes in cellular protein, which is similarly affected by both thiamin and oxythiamin, but in T98G cells only. As a result, independent of protein level, the thiamin antagonist oxythiamin increases cellular NAD(P)H:resazurin oxidoreductase in both T98G and U87 cells in the time- and concentration-dependent manner, while thiamin even slightly decreases the activity (Figure 1).

### 3.2. Biochemical Analysis of the Thiamin and Oxythiamin Effects in Cell Extracts of T98G and U87 Cells

The known anticoenzyme action of oxythiamin *in vivo* [15] was confirmed by assays of both the 2-oxoglutarate dehydrogenase (OGDH) and pyruvate dehydrogenase (PDH) complexes, which exhibited strong inhibition after incubation with 5 mM oxythiamin. However, unlike the OGDH assay, the NAD^+^-dependent assay of PDH complex in cell extracts is perturbed by lactate dehydrogenase. Using the color reaction of the PDH dipstick assay (Abcam) did not enable efficient study of the time and concentration dependencies. Hence, OGDH was used as the marker of cellular action of oxythiamin on the ThDP-dependent enzymes (Figure 2).

Figure 2 also indicates that several enzymes metabolically linked to the ThDP-dependent ones, responded to cellular treatments with thiamin and oxythiamin. These were the malic enzyme linked to PDH through the pyruvate production, MDH linked to PDH and OGDH through the oxaloacetate production and malate/2-oxoglutarate transporter, correspondingly, and GDH linked to OGDH through the 2-oxoglutarate production. Similar to the changes in protein level (Figure 2A–D), the enzymatic responses were also cell-specific. In particular, OGDH and GDH were mostly downregulated in U87 cells, but exhibited significant up-regulation in T98G cells. In addition, malic enzyme was unreactive in U87 cells, but changed its activity in T98G cells. The biochemical differences between T98G and U87 cells were also well seen by following the levels of the enzyme activities during cell incubation in the control medium (DMEM with 4.5 g/L glucose, supplemented with 10% FCS, 2 mM GlutaMAX™ and antibiotics). As seen from Table 1, between 5 h and 24 h of the incubation, both cell lines increased about 2-fold their GDH and MDH activities. However, this was accompanied by the cell-specific increase in OGDH (app. 2-fold) in U87 cells and up-regulation of malic enzyme (app. 6-fold) in T98G cells. Thus, the cell-specific responses of the central metabolic enzymes in T98G and U87 cells were observed both upon the thiamin/oxythiamin treatments (Figure 2) and incubation of the non-treated cells (Table 1). Malic enzyme was much more reactive in all treatments of T98G compared to U87 cells. Also, the high level of OGDH and GDH in U87 *vs.* T98G cells after 24 h of incubation in control medium (Table 1) coincided with a less expressed up-regulation of these enzymes in U87 *vs.* T98G cells responding to oxythiamin (Figure 2).

Changes in cellular protein under the oxythiamin treatment of T98G cells (Figure 2B) correlated with a more complex biochemical responses to the treatment of T98G cells, compared to U87 cells, where protein did not change (Figure 2D). Almost complete inhibition of OGDH by oxythiamin was observed in U87 cells after 24 h incubation of cells with a high (5 mM) concentration of oxythiamin (Figure 2H). However, in T98G cells OGDH was significantly inhibited after 5 h incubation with 5 mM oxythiamin, but in 24 h the activity was restored to the control level (Figure 2F). At a low oxythiamin concentration (0.05 mM) the up-regulation of OGDH after 24 h incubation could be seen in both cell lines, yet the amplitude of the activity increase was higher in T98G *vs.* U87 cells. The time- and concentration-dependent changes in the OGDH activity thus indicate that the oxythiamin-dependent enzyme inhibition is addressed by the OGDH up-regulation, which is much more pronounced in T98G cells.

In response to oxythiamin, T98G cells also demonstrated the ability to up-regulate GDH and malic enzyme (Figure 2J,R), which did not occur in U87 cells (Figure 2L,T). In fact, oxythiamin decreased GDH in U87 cells, while malic enzyme remained unchanged (Figure 2L,T). Similar to OGDH, GDH and malic enzyme showed biphasic changes in T98G cells. However, the sequence of events was opposite. In contrast to OGDH, up-regulation of GDH and malic enzyme preceded their down-regulation. As a result, in T98G cells, OGDH and metabolically linked enzymes exhibited complementary changes. Initial inhibition of OGDH by oxythiamin occurred along with activation of GDH and malic enzymes, whereas the inhibition-induced up-regulation of OGDH was accompanied by decreases in GDH and malic enzyme (Figure 2). MDH was up-regulated in response to oxythiamin in both T98G and U87 cells, although the maximal amplitude of the activation observed was about 2-fold higher in the former (40%) compared to the latter (20%) (Figure 2N,P).

**Table 1:** Total protein and enzyme activities in T98G and U87 cells during incubation in cell culture medium. The data are presented as mean ± SEM. Statistically significant differences (*p* < 0.05) between 5 h and 24 h (**a**—T98G 5 h *vs.* T98G 24 h; **b**—U87 5 h *vs.* U87 24 h) or between cells at the same growth time (**c**—T98G 5 h *vs.* U87 5 h; **d**—T98G 24 h *vs.* U87 24 h) were determined by one-way ANOVA with Tukey’s Multiple Comparison Test. Statistically significant up-regulation of the enzymes in each cell line during the incubation is shown in bold.

Cell Line	T98G		U87	
Time in Medium	5 h	24 h	5 h	24 h
**Protein, mg/mL** (c,d)	1.73 ± 0.08	1.81 ± 0.00	1.33 ± 0.01	1.34 ± 0.00
**Activity, μmol/(min∙mg)**				
**OGDH** (b,d)	0.05 ± 0.01	0.03 ± 0.00	0.07 ± 0.02	**0.16 ± 0.01**
**GDH** (b)	0.016 ± 0.003	0.032 ± 0.003	0.030 ± 0.002	**0.052 ± 0.012**
**MDH** (a,b,c,d)	7.8 ± 0.4	**13.3 ± 0.3**	10.6 ± 0.6	**18.7 ± 0.9**
**Malic** (a,d)	0.09 ± 0.01	**0.57 ± 0.01**	0.06 ± 0.01	0.11 ± 0.02

It is remarkable that treatment of T98G cells with thiamin reproduced responses of MDH and malic enzymes to oxythiamin. Also the thiamin effects on OGDH and GDH were similar to the oxythiamin effects, yet not reaching statistical significance. The only difference between the action of thiamin and oxythiamin on T98G cells was observed at a low dose of thiamin (0.05 mM for 5 h). After this treatment T98G cells exhibited a transient inhibition of MDH by thiamin, which disappeared after 24 h of incubation (Figure 2M). In U87 cells, similarity of the thiamin and oxythiamin actions was seen from their effects on GDH and MDH (Figure 2K–P).

As a result, response of OGDH to the treatment with either thiamin or oxythiamin is translated to the metabolically linked enzymes affiliated with the TCA cycle (MDH, GDH, malic enzyme). Overall, the response of the non-ThDP-dependent enzymes to (oxy)thiamin is more pronounced in the cells (T98G) which can significantly up-regulate the ThDP-dependent OGDH under this condition. In its turn, the amplitude of the OGDH up-regulation in the presence of oxythiamin (Figure 2) correlates with the cell-specific level of the enzyme activity (Table 1.) In cells with a low OGDH activity (T98G) the OGDH up-regulation is higher than in cells with a high OGDH activity (U87). Finally, the biphasic and well-expressed enzymatic responses of T98G cells to both thiamin and oxythiamin are associated with a decrease in cellular protein (Figure 2).

### 3.3. Changes in Cellular Enzyme Activities, Accompanying the Oxythiamin-Induced Increase in Cellular NAD(P)H:Resazurin Oxidoreductase

**Figure 3:**
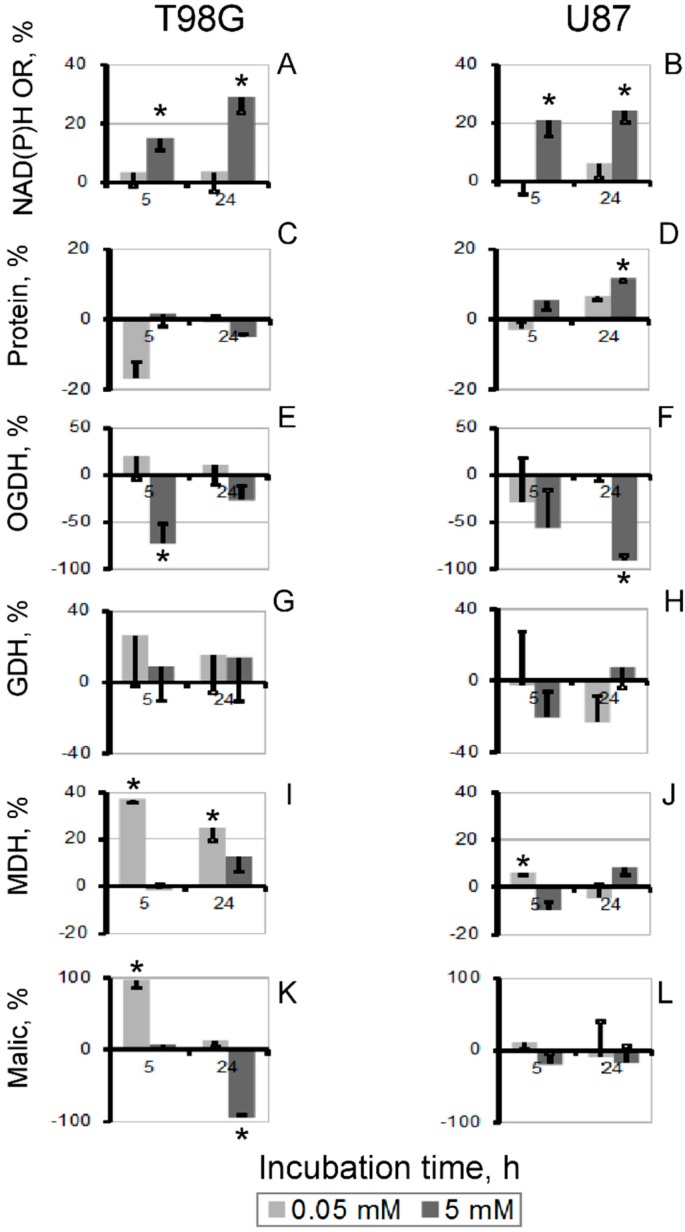
Differences in the thiamin and oxythiamin effects on NAD(P)H:resazurin oxidoreductase (NAD(P)H OR) (**A**,**B**), protein (**C**,**D**), and tested enzymatic activities (**E**–**L**) in T98G (left panel) and U87 (right panel) cells. The data of Figure 1 and Figure 2 were used to calculate the difference between the indicated parameters in the oxythiamin- and thiamin- treated cells, represented as mean ± SEM, %. SEM was calculated after summation of StDev in each experimental group. Significance of the difference between the thiamin and oxythiamin effects (* *p* < 0.05) was determined by Student’s *t*-test.

In contrast to the similar, but cell-specific action of thiamin and oxythiamin on protein level, the increase in the NAD(P)H:resazurin oxidoreductase activity was oxythiamin-specific and observed in both T98G and U87 cells (Figure 1). To reveal the associated changes in the tested metabolic enzymes producing NAD(P)H, we subtracted the thiamin effects on the enzymes from those of oxythiamin. The differential comparison presented in Figure 3 reveals that the major difference in the enzymatic activities, associated with increased NAD(P)H:resazurin oxidoreductase (Figure 3A,B), is decreased cellular OGDH activity. Although the decrease in the OGDH activity between the oxythiamin and thiamin-treated cells does not always reach statistical significance (Figure 3E,F), it is nevertheless consistently observed under all conditions revealing increased NAD(P)H:resazurin oxidoreductase, i.e., 5 h and 24 h incubation with 5 mM compounds (Figure 3A,B). In T98G cells, which may significantly up-regulate OGDH in response to oxythiamin and therefore exhibit the lowest (about 20%) decrease in the OGDH activity between the oxythiamin- and thiamin-treated cells (Figure 3C, 5 mM for 24 h), significant decrease in activity of malic enzyme was associated with increased NAD(P)H:resazurin oxidoreductase (Figure 3K, 5 mM for 24 h). In view of the inhibition by oxythiamin of both OGDH and PDH, down-regulation of the pyruvate-producing malic enzyme obviously manifests response of T98G cells to the pyruvate accumulation.

### 3.4. Increase in Cellular NAD(P)H:Resazurin Oxidoreductase by the Site-Directed Inhibitors of Mitochondrial 2-Oxo Acid Dehydrogenases

**Figure 4:**
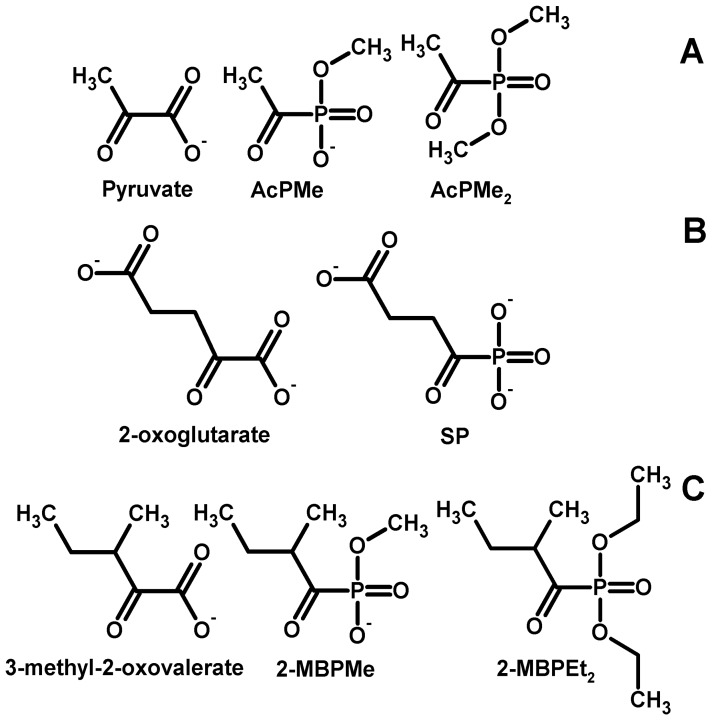
Structures of the 2-oxo acids and their phosphonate analogs used in this work. (**A**) Pyruvate and its analogs: methyl ester of acetyl phosphonate (AcPMe) and dimethyl ester of acetyl phosphonate (AcPMe_2_); (**B**) 2-oxoglutarate and its analog, succinyl phosphonate (SP); (**C**) 3-methyl-2-oxo-valerate (“ketoisoleucine”) and its analogs, methyl ester of 2-methylbutyryl phosphonate (2-MBPMe) and diethyl ester of 2-methylbutyryl phosphonate (2-MBPEt_2_).

**Figure 5:**
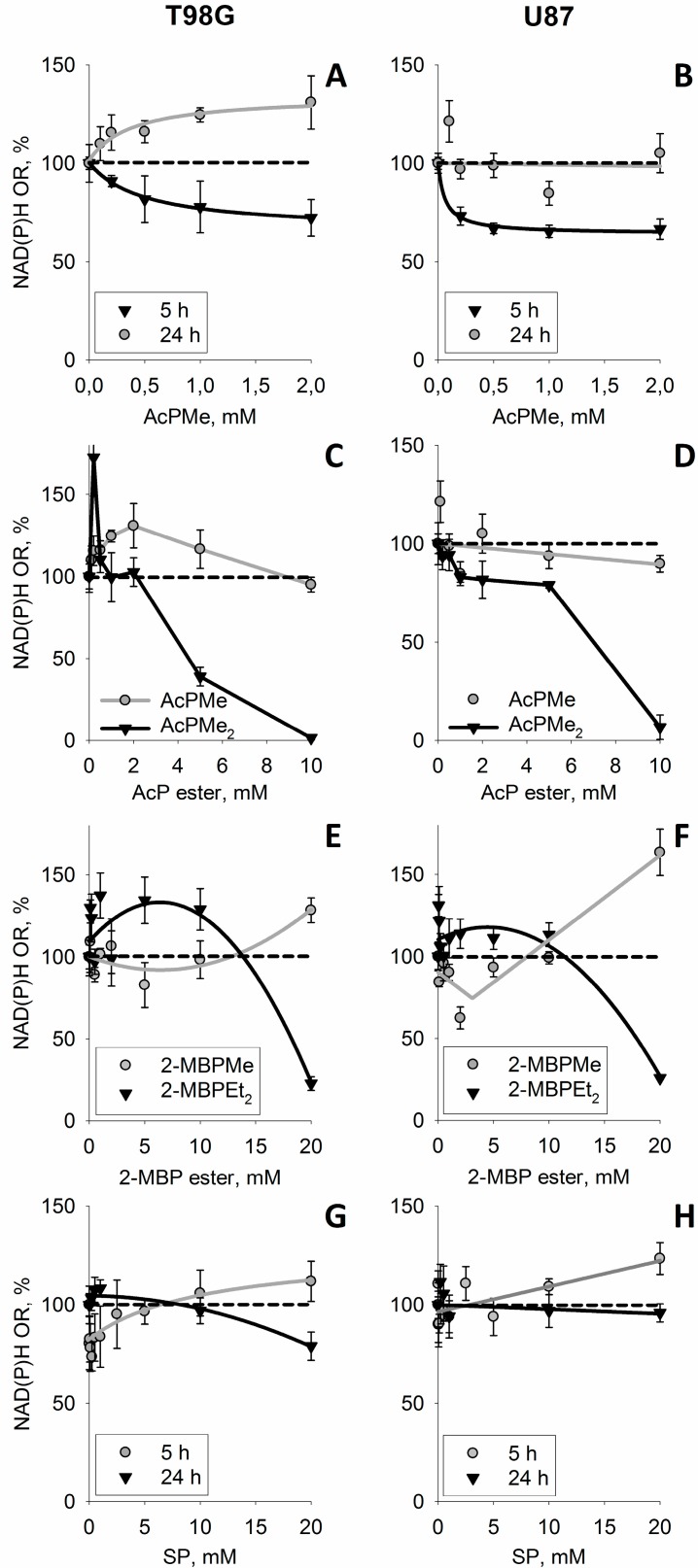
The time- and concentration-dependent changes in the NAD(P)H:resazurin oxidoreductase (NAD(P)H OR) of T98G (left panel) and U87 (right panel) cells due to the phosphonate analogs of pyruvate (**A**–**D**), branched-chain 2-oxo acids (**E**,**F**) and oxoglutarate (**G**,**H**). The phosphonate analogs were incubated with cells in HBSS (**A**–**D)** or DMEM (**E**–**H**). Different incubation times (**A**,**B**,**G**,**H**) or analogs (**C**–**F**) are indicated in the legends on the graphs. When not indicated (**C**–**H**), the incubation time was 24 h. Structures and abbreviations of the phosphonate analogs used are given in Figure 4.

Complex changes in both the ThDP-dependent and ThDP-independent enzymes due to the action of (oxy)thiamin (Figure 2) could include the non-coenzyme action of thiamin as metabolic regulator [16]. To distinguish this action from the coenzyme role of thiamin and confirm the leading role of the mitochondrial ThDP-dependent enzymes in the oxythiamin-induced increase of cellular NAD(P)H:resazurin oxidoreductase (Figure 1), we took advantage of the phosphonate analogs of 2-oxo acids as specific site-directed inhibitors of the mitochondrial ThDP-dependent 2-oxo acid dehydrogenases [15]. Figure 4 presents the structures of the employed phosphonate analogs of pyruvate (A), 2-oxoglutarate (B), a branched-chain 2-oxo acid 3-methyl-2-oxovalerate formed from isoleucine (C) and their abbreviations used. The mechanism of action of the phosphonates implies specific inhibition of mitochondrial ThDP-dependent dehydrogenases of pyruvate, 2-oxoglutarate and branched-chain 2-oxo acids by respective substrate analogs [15]. To check if the inhibition of the particular 2-oxo acid dehydrogenases could mimic the oxythiamin-induced increase of cellular NAD(P)H:resazurin oxidoreductase (Figure 1), we screened the impact on different 2-oxo acid analogs on the reaction (Figure 5). Indeed, transient increases in cellular NAD(P)H:resazurin oxidoreductase could be revealed, with their kinetics and amplitude dependent on the analog structure, concentration and incubation time. For instance, Figure 5A,B show that, after a short incubation time (5 h), AcPMe induced a concentration-dependent decrease in cellular NAD(P)H:resazurin oxidoreductase in both the T98G and U87 cells. Yet after the incubation time increased to 24 h, no more decrease was observed. Instead, the NAD(P)H:resazurin oxidoreductase of T98G cells exhibited a concentration-dependent increase. Between 5 h and 24 h of incubation with AcPMe, U87 cells did not increase their NAD(P)H:resazurin oxidoreductase over the control level, but the increase after 24 h was observed when compared to 5 h of incubation with the inhibitor (Figure 5B). As a result, after 24 h incubation with 2 mM AcPMe in the medium, cellular NAD(P)H:resazurin oxidoreductase of T98G cells increased app. 1.7-fold, compared to the reaction rate after 5 h with 2 mM AcPMe (Figure 5A). In U87 cells the increase corresponded to 30% of the control value (Figure 5B).

In spite of the time-dependent increase in the NAD(P)H:resazurin oxidoreductase in the presence of AcPMe (Figure 5A,B), instability of cellular proliferation under these conditions was seen when the concentration interval was expanded. The negative effect of the PDH inhibition on viability becomes especially obvious when a membrane-permeable uncharged analog AcPMe_2_, which gets into cells more efficiently and is transformed to the inhibitory species (AcPMe and the fully de-esterified phosphonate) by intracellular esterases [15], is added to cell medium (Figure 5C,D). Obviously, the membrane permeability of uncharged AcPMe_2_, compared to the negatively charged AcPMe, increases intracellular concentration of the inhibitory species, leading to more pronounced changes in cellular NAD(P)H:resazurin oxidoreductase over the same time and concentration interval (Figure 5C,D). Remarkably, also upon treatment with AcPMe_2_ one could observe an initial increase in the NAD(P)H:resazurin oxidoreductase. Similar to AcPMe, the increase is more expressed in T98G than in U87 cells. Compared to charged AcPMe, the AcPMe_2_-induced increase is of a higher amplitude and is observed at lower concentrations. These features of the AcPMe_2_ action are associated with strong destabilization of the system. That is, the temporary increase in the NAD(P)H:resazurin oxidoreductase is followed by an irreversible loss of the activity upon incubation with the PDH inhibitor (Figure 5C). Under the same conditions, the more resistant U87 cells do not increase the NAD(P)H:resazurin oxidoreductase as strong as T98G cells do. This is accompanied by a well-expressed intermediary plateau in dependence of the NAD(P)H:resazurin oxidoreductase of U87 cells on AcPMe_2_ concentration (Figure 5D).

Similar temporary increases in the NAD(P)H:resazurin oxidoreductase were observed for T98G and U87 cells upon incubation in the presence of the branched-chain 2-oxo acid dehydrogenase (BCDH) inhibitors 2-MBPMe and 2-MBPEt_2_ (Figure 5C). Because BCDH takes part in oxidation of the branched chain amino acids, the action of these inhibitors was tested in rich (DMEM) medium comprising amino acids. A less significant contribution to the viability of BCDH, compared to the central metabolic role of PDH, is in good accordance with the higher resistance of cells to the inhibitors of BCDH (Figure 5E,F), compared to those of PDH (Figure 5C,D). However, at a fixed incubation time, the concentration dependence of the NAD(P)H:resazurin oxidoreductase exhibits a characteristic difference for the charged (2-MBPMe) and uncharged (2-MBPEt_2_) inhibitor. While at 20 mM 2-MBPMe the NAD(P)H:resazurin oxidoreductase of T98G cells is still significantly increased, 20 mM 2-MBPEt_2_ already causes the viability loss, obvious as decreased NAD(P)H:resazurin oxidoreductase. However, at lower concentrations of 2-MBPEt_2_ a transient increase may be observed also with this analog.

The time and concentration dependencies of cellular NAD(P)H:resazurin oxidoreductase on the OGDH inhibitor SP are shown in Figure 5G,H. Similar to earlier studies in neuronal cultures, the response of the NAD(P)H:resazurin oxidoreductase to the OGDH inhibitor is not much expressed, presumably due to the efficient shunt of the SP block by oxidation of the branched-chain 2-oxo acids [17,18]. However, also with this inhibitor one could distinguish a slight concentration-dependent increase in the NAD(P)H:resazurin oxidoreductase at a low incubation time of 5 h, transformed into a decrease after 24 h of inhibition by the same concentrations of SP. Unlike the other phosphonates used, triethylated SP is of a lower chemical stability, which did not allow us to perform the comparison, as in Figure 5C–F.

Thus, the primary decreases in cellular NAD(P)H:resazurin oxidoreductase, which could correspond to decreased NADH production by the 2-oxo acid dehydrogenases (Figure 5A,B, 5 h), are rarely obvious. They are of a low amplitude and/or masked by a secondary increase in the NAD(P)H:resazurin oxidoreductase. The increase is similar to the effect on the oxidoreductase of the *in vivo* inhibitor of all ThDP-dependent enzymes, oxythiamin. However, action of the phosphonates allows one to see that the increase is only temporary, with strong and/or persistent impairment of the mitochondrial ThDP-dependent 2-oxo acid dehydrogenases by the phosphonates expectedly decreasing the NAD(P)H:resazurin oxidoreductase. Thus, cellular viability is impaired by the phosphonates, despite the temporary increase in the NAD(P)H:resazurin oxidoreductase.

### 3.5. Discussion

#### 3.5.1. Inhibition of Cellular NAD(P)H Producers May Increase NAD(P)H:Resazurin Oxidoreductase

**Figure 6:**
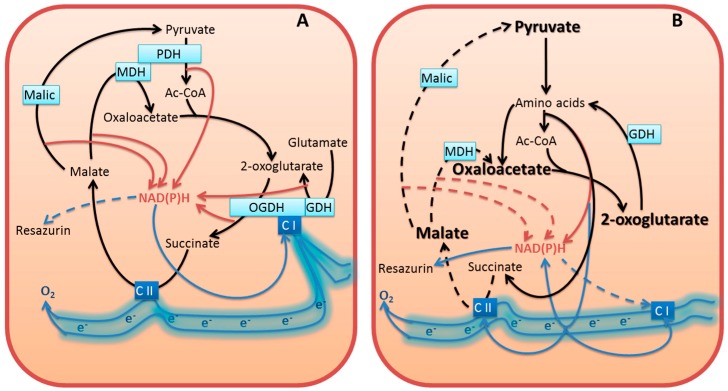
Potential changes in the electron flow to resazurin in normal (**A**) and perturbed (**B**) metabolism, based on the enzyme activity data after 24 h incubation of cells with oxythiamin (Figure 2 and Figure 3, 24 h). Due to transamination of the 2-oxo acids which accumulate upon inhibition of the 2-oxo acid dehydrogenases [17,22], amino acids become substrates for oxidation, generating NAD(P)H, FADH_2_, ubiquinone QH_2_ [23]. These compounds are in redox equilibrium with other acceptors of the electron transport chain, such as redox sites of Complexes I and II (C I and C II). Driven by inhibition of the 2-oxo acid dehydrogenases, the pathophysiological increase in the amino acid oxidation (e.g., through the GABA shunt and proline oxidation) may increase side reactions, including the resazurin reduction, due to unphysiological electron flux. The substrate-dependent NAD(P)H production is depicted by brown arrows. Other electron transfers are shown by blue arrows. The enzymes assayed in Figure 2 are presented within the light-blue rectangles. Well-characterized enzymatic complexes of the TCA cycle enzymes are shown by merged rectangles. Electron-transport chain and its Complexes I (NADH:ubiquinone oxidoreductase) and II (succinate dehydrogenase) are shown in blue. For the sake of readability, the schemes omit cellular compartmentation of the processes, assuming exchange of the intermediates through the substrate/electron shuttles, and show only the relevant intermediates, reactions, and protein partners. In particular, (i) the electron flow is schematically shown as floating electrons instead of separate redox active sites of the electron transport chain, as the distribution of electrons between the sites would depend on specific conditions and metabolism; (ii) to manifest the consequences of the PDH and OGDH inhibition, the enzymes are omitted from (B). The decreased activity of malic enzyme concomitant to elevated NAD(P)H:resazurin oxidoreductase (Figure 3K) is shown by dashed line in (B). Dashed lines of the reactions, catalyzed by the mitochondrial MDH and Complex II refer to inhibition of the indicated transformations upon accumulation of oxaloacetate and malate. Oxaloacetate increases due to intensified transamination of 2-oxoglutarate as a consequence of the OGDH inhibition. Malate increases due to decrease in malic enzyme and/or inhibition of malate oxidation by the product oxaloacetate. Additional inhibition of the succinate oxidation to fumarate by Complex II may be due to alternative generation of fumarate from the 2-oxoglutarate-stimulated oxidation of tyrosine [17]. Dashed line from NAD(P)H to Complex I refers to decreased NADH from the physiological NADH suppliers (PDH and the TCA cycle enzymes) of Complex I. See text for further discussion.

In this work, we show that the inhibitors of cellular NAD(P)H producers, oxythiamin and phosphonate analogs of 2-oxo acids, may cause a paradoxical increase in cellular NAD(P)H:resazurin oxidoreductase (Figure 1 and Figure 5). Our data suggest that diminished mitochondrial oxidation of NAD(P)H in the 2-oxo acid dehydrogenase-comprising metabolons may cause an artifactual increase in the electron flux to artificial dyes in the NAD(P)H-based viability assay. This is important to take into account when considering cellular NAD(P)H:resazurin oxidoreductase as an indicator of cellular viability. The commercially available viability tests using this reaction [19,20,21] are based on the fact that proliferation requires NAD(P)H for biosynthesis and antioxidant defense, with the side reaction of the NAD(P)H:resazurin oxidoreductase being a measure of cellular reducing power dependent on the NAD(P)H level (Figure 6A). However, our study demonstrates that under conditions of impaired metabolism an increase in cellular NAD(P)H:resazurin oxidoreductase may be observed, which does not mean increased NAD(P)H available for cell proliferation (Figure 6B). Below, we consider possible mechanisms of the processes involved (Figure 6), suggested by the data obtained in this and other works.

Resazurin, or Alamar Blue, belongs to the tetrazolium dyes, which include a wide variety of redox compounds changing their spectral properties upon reduction [9]. Compared to the older assays of cellular viability employing 3-(4,5-dimethylthiazol-2-yl)-2,5-diphenyltetrazolium (MTT), resazurin assays (Alamar Blue assay of Invitrogen, CellTiterBlue assay of Promega) possess several advantages. Both the oxidized (resazurin) and reduced (resorufin) forms of this dye are water-soluble and non-toxic, enabling reaction monitoring in live cells. After diffusion into cells, resazurin is reduced by cytosolic and mitochondrial dehydrogenases, such as flavin mononucleotide and flavin adenine dinucleotide dehydrogenases, nicotinamide adenine dinucleotide (phosphate) dehydrogenases, aldehyde reductase, alcohol dehydrogenase, NAD(P)H-dependent quinone reductase, flavin reductase and diaphorase [21,24,25,26]. In contrast to MTT (E_0_ = −110 mV), the standard redox potential of resazurin (E_0_ = +380 mV) indicates that resazurin may also be reduced at the expense of cytochromes (E_0_ from +80 to +290 mV) [25,27]. Although the potentials indicate that increased reduction of cytochromes allows for the resazurin reduction, which would not occur with MTT, kinetic competence of resazurin to directly accept electrons from cytochromes was not shown.

Most probably, the resazurin reduction is mediated by the NAD(P)H-dependent oxidoreductases. However, dependent on the electron distribution between different redox components, different rates of the reduction may also be due to different sets of the resazurin-reducing oxidoreductases involved under different conditions (Figure 6). This may lead to the paradoxical observation presented in Figure 1, where thiamin and oxythiamin, whose metabolic action is known to increase and decrease the NAD(P)H levels, correspondingly [28], unexpectedly cause the opposite increments in the NAD(P)H:resazurin oxidoreductase activities. That is, thiamin decreases, and oxythiamin increases the activity (Figure 1). Rather than manifesting more NAD(P)H available for cellular biosynthesis and antioxidant defense (Figure 6A), the finding apparently indicates changes in the substrate and electron flow (Figure 6B). In particular, the changes may result in stimulation of the resazurin reductases coupled to the mitochondrial electron transport chain.

#### 3.5.2. Compensation for Impairment of Metabolic Checkpoints Perturbs the Physiological Flux of Substrates and Electrons

The ThDP-dependent dehydrogenases of the 2-oxo acids occupy metabolic checkpoints, and are tightly regulated [15,29]. In particular, the evolutionary optimized supramolecular structures control the substrate and electron flow in normal metabolism. For instance, OGDH complex, which is often rate-limiting for the TCA cycle flux (reviewed in [29,30]), interacts with both GDH [31,32] and mitochondrial Complex I of the respiratory chain [33,34]. As a result, the overall NADH generation in the TCA cycle depends on the mutually regulated capacity of the physically interacting OGDH and Complex I. The supramolecular structure-supported control of the NADH supply to Complex I should prevent undesirable electron overflow and ensuing side reactions of the electron transport chain, such as production of reactive oxygen species (ROS). When 2-oxoglutarate dehydrogenase is inactivated and/or dysregulated, no decrease in cellular reducing power is obvious from testing the NAD(P)H-dependent electron transfer to resazurin (Figure 5G,H), [17] because of compensatory reactions discussed below. However, the NAD(P)H production in these compensatory reactions is not regulated as tightly as it is in the supramolecular structures which evolved to support normal metabolism. As a result, normal substrate and electron flow may be perturbed and side reactions activated. This would decrease viability even when cellular reducing potential does not decrease. Indeed, increased inhibition of OGDH increases ROS production [35,36]. Also increased proline oxidation is known to increase ROS [23]. Oxaloacetate may accumulate under the oxythiamin-dependent inhibition of PDH and OGDH (Figure 6B), because the OGDH inhibition increases transamination of 2-oxoglutarate with aspartate, while the inhibition of PDH may decrease efficiency of the oxaloacetate condensation with acetyl-CoA. The oxaloacetate accumulation inhibits physiological direction of the mitochondrial MDH reaction (depicted by the dashed lines in Figure 6B). The observed MDH increase in the *in vitro* assay testing the thermodynamically favored reduction of oxaloacetate to malate (Figure 2) may be a compensatory response to inefficient malate oxidation under conditions of the oxalacetate accumulation. Probably, the effect involves the interaction of mitochondrial MDH with the cytochrome *bc*_1_ complex, which was shown to activate particularly the backward reaction of the oxaloacetate reduction by the MDH, as well as reduction of *bc*_1_ complex [37]. On the other hand, oxaloacetate is a strong inhibitor of Complex II [38]. Thus, perturbation in the TCA cycle due to the inhibition of the NADH-producing 2-oxo acid dehydrogenases is translated to the electron transport chain. Also the malate-aspartate shuttle for oxidation of cytosolic NAD(P)H by mitochondria is perturbed when mitochondrial oxaloacetate is accumulated. As a result, the NAD(P)H generated at metabolic impairment cannot be properly oxidized, which may induce increased electron flow to the side reaction of resazurin reduction (Figure 6B). This is observed under inhibition of the ThDP-dependent enzymes by oxythiamin (Figure 1) or particular ThDP-dependent mitochondrial dehydrogenases of 2-oxo acids (Figure 5) by the phosphonate analogs of their substrates (Figure 4).

Overall, the data obtained show that interactive nature of metabolic reactions allows cells to more or less efficiently compensate for the inhibition of particular NAD(P)H producers, but this is accompanied by significant changes in cellular metabolome and biosynthesis. For instance, action of SP on primary neurons in culture showed the same poor response of the NAD(P)H:resazurin oxidoreductase to the OGDH inhibition, as observed with glioblastoma cells in Figure 5G,H, yet dramatic changes in neuronal metabolome were observed under these conditions [17]. In other studies, unaltered pyridine nucleotide content was shown upon antisense inhibition of OGDH expression, in spite of considerable reduction in the OGDH activity and mitochondrial respiration rates [39]. When OGDH was almost completely inhibited by SP [40], the absolute levels of NADPH and NADH did not decrease, but the redox ratio of NAD(P)H/NAD(P)^+^ decreased due to elevated NAD(P)^+^. Remarkably, despite inhibition of the NADH-producing OGDH, a higher decrease was observed for NADPH/NADP^+^ (35%), compared to NADH/NAD^+^ (25%), providing additional evidence for the changed electron distribution (Figure 6B) rather than direct effect of the OGDH inhibition on the NADH levels. Obviously, the changed electron flow is defined by the OGDH inhibition-dependent switch to the amino acid oxidation [17,18,22,41]. Because of unequal significance of particular amino acids as energy sources, the total amino acid pool upon the OGDH inhibition usually increases, with decreasing protein content (reviewed in [15]). In contrast, upon antisense inhibition of the upstream TCA cycle NADH producer, the mitochondrial NAD^+^-dependent isocitrate dehydrogenase, the amino acid pool did not change, while the protein content increased [42]. This different effect on the amino acids and protein correlated with the different changes in the pyridine nucleotide pool. While the OGDH impairment decreased NAD(P)H/NAD(P)^+^ due to increased NAD(P)^+^ [40], inhibition of mitochondrial NAD^+^-dependent isocitrate dehydrogenase decreased NADPH and NADH without statistically significant changes in NAD(P)H/NAD(P)^+^ [42]. Moreover, when cytosolic NADP^+^-reducing isocitrate dehydrogenase was inhibited, NADH decreased more than NADPH, leading to the statistically significant decrease in NADH/NAD^+^ only. The amino acid pool decreased in this case, but protein level did not change [43]. Thus, inhibition of the cytosolic and mitochondrial NAD(P)H production from isocitrate and 2-oxoglutarate exhibits coupled differences in their effects on the pyridine nucleotide pool and amino acid/protein levels. Increased NAD(P)^+^ at constant NAD(P)H is associated with increased amino acid pool and decreased protein. This is observed upon OGDH inhibition [40]. In contrast, constant NAD(P)^+^ at decreased NAD(P)H is associated with no increase in the amino acid pool and no decrease in protein. This is observed upon inhibition of the cytosolic [43] and TCA cycle [42] isocitrate dehydrogenases. The data indicate that specific changes in the substrate and electron fluxes depend on metabolic impairments. Obviously, the changes are due to the compensatory pathways, which are activated to support homeostasis by available substrates. Condition-dependent metabolomes and supramolecular complexes, such as those between OGDH, MDH, GDH and/or affiliated enzymes [12,31,32,33,34,44,45], stimulate oxidation of intermediates through different pathways. For instance, MDH may form a ternary complex either with OGDH and aspartate transaminase, or with PDH and citrate synthase [44]. The former is important for function of malate-aspartate shuttle of electrons between cytoplasm and mitochondria, while the latter supports citrate synthesis. The cross-talk between the mitochondrial MDH and *bc_1_* complex of electron transport chain may participate in organization of the oxaloacetate-dependent electron flow [37]. In the supramolecular structures, regulation of enzymatic activities may occur through the protein-protein interactions, induced binding of secondary messengers and/or post-translational modifications. All these factors along with the transcriptional regulation may contribute to activation of the alternative pathways of NAD(P)H generation and electron flow upon intracellular inhibition of the ThDP-dependent enzymes by oxythiamin (Figure 6B).

#### 3.5.3. Dysregulated Metabolism Increases Cellular Side Reactions

As shown in the previous section, dysregulated metabolism may increase the flow of electrons to resazurin. Metabolic impairments are also known to increase another cellular side reaction, the 1e^−^ reduction of oxygen with ROS formation. Indeed, like the oxythiamin-induced increase in the NAD(P)H:resazurin oxidoreductase (Figure 1), increased ROS were observed upon treatments with the thiamin antagonists oxythiamin [28] and pyrithiamin [46]. While the NAD(P)H:resazurin oxidoreductase increased upon blocking the NAD(P)H production (Figure 1 and Figure 5), ROS increased upon inhibition of the known ROS producers, such as Complex 1 and OGDH [35,36,47]. The consistent increase in MDH activity by oxythiamin, observed in our experiments (Figure 2J,L), may point to increased ROS production, because oxidative stress, known to occur upon the OGDH inhibition *in vivo* [46,48] and *in situ* [35,36], was associated with increased expression of MDH [49].

Taken together, these findings support the assumption that dysregulation of the mitochondrial electron transport chain due to sub-optimal supply of electrons from the reactions compensating for the inhibition of the 2-oxo acid dehydrogenases, may increase cellular side reactions, such as ROS generation or resazurin reduction.

#### 3.5.4. Similarity between the Action of Thiamin and Oxythiamin on Glioblastoma Cells

Thiamin is a coenzyme of central metabolism which is efficiently pumped into cancer cells, supposedly to increase the transketolase-dependent NAD(P)H and ribose production, necessary for intense proliferation [15,50,51,52]. Antimetabolic action of the catalytically inactive thiamin analogs has therefore been used to kill cancer cells (reviewed in [15]). The antimetabolic action of oxythiamin is known to be mediated by its *in vivo* diphosphorylation in the thiamin diphosphokinase reaction to oxythiamin diphosphate. OGDH forms the tightest complex with ThDP, compared to other ThDP-dependent enzymes, [29]. The tight ThDP binding makes OGDH the least susceptible to substitution of the coenzyme by catalytically inactive oxythiamin diphosphate. This implies that inhibition of cellular OGDH by oxythiamin occurs when ThDP in other binding active sites has been already substituted by the catalytically inactive coenzyme analogs. Owing to this, OGDH is a suitable marker of the oxythiamin saturation of all ThDP-dependent enzymes in cells.

A comparative study of the thiamin and oxythiamin effects on the two glioblastoma cell lines reveals surprising similarities in the action of both compounds, which may, however, be differently expressed in the two cells lines. First of all, both thiamin and oxythiamin significantly decrease total protein in T98G cells (Figure 2A). Second, in T98G cells both thiamin and oxythiamin may up-regulate GDH, MDH and malic enzyme, although the thiamin-dependent up-regulation of GDH (Figure 2E) does not reach statistical significance. In U87 cells, both thiamin and oxythiamin decrease GDH and increase MDH. Thus, thiamin and oxythiamin demonstrate common cell-specific effects.

The action of the OGDH inhibitor oxythiamin on the protein level in T98G cells (Figure 2A,B) aligns with our previous studies discussed above, when the OGDH inhibition decreased protein level in both plant and animal cells [17,22]. This is supposedly due to increased oxidation of amino acids, shunting the OGDH block in the TCA cycle. These include the branched-chain amino acids, tyrosine and lysine [17,18]. A similar effect on protein level of the OGDH activator thiamin, observed in the current work (Figure 2A), may be also due to increased oxidation of amino acids in the TCA cycle after the rate-limiting OGDH is activated by thiamin. In this case, however, the intensified catabolism of amino acids and proteins may be due to the OGDH-mediated oxidation of the amino acids of the glutamate group. The increased flux through the TCA cycle in T98G cells is supported by elevated generation of the TCA cycle intermediates in the activated MDH and GDH reactions, activation of the malic enzyme generating pyruvate (Figure 2), and expected activation of PDH by thiamin. Remarkably, no decrease of cellular protein in U87 cells is accompanied by a significant down-regulation of GDH in these cells. Thus, increased provision of 2-oxoglutarate and pyruvate for the TCA cycle may stimulate the amino acid degradation through the thiamin-activated OGDH and PDH. When the 2-oxo acid dehydrogenases are inhibited by oxythiamin, compensatory changes may involve increased oxidation of lysine and tyrosine, producing the TCA cycle substrate acetyl-CoA and intermediate fumarate, correspondingly. As a result, the protein levels decrease after treatments of T98G cells with either thiamin or oxythiamin, although different amino acids may be depleted in the two cases.

The up-regulation of OGDH and related enzymes in T98G cells (Figure 2) is more expressed after a long (24 h) exposure to a low (0.05 mM) concentration of oxythiamin. This is obviously due to a lower inhibitory effect of oxythiamin on OGDH under these conditions. The OGDH up-regulation over the control values reaches statistical significance in T98G cells only, where it also can be seen after 24 h incubation with 5 mM oxythiamin, if compared to the enzyme inhibited at this concentration after 5 h (Figure 2F). U87 cells exhibit analogous behavior at a low (0.05 mM) concentration of oxythiamin only (Figure 2H). These findings point to the superposition of the oxythiamin-induced inhibition and up-regulation of OGDH in response to this inhibition, with the up-regulation more expressed in T98G than U87 cells. The differential regulation of the enzymes in T98G and U87 cells coincides with the effect of (oxy)thiamin on protein level in T98G cells only. 

Thus, the data obtained indicate that the similar action of thiamin and oxythiamin on protein level may be explained by metabolic interaction between the ThDP-dependent and ThDP-independent enzymes. Our recent study provided evidence that these metabolic links are strengthened by the non-coenzyme action of thiamin as an allosteric regulator and/or mediator of regulatory acetylation [16]. It is therefore probable that the similarities between the thiamin and oxythiamin actions on the metabolic state of a cell (Figure 2) may to a certain extent rely on the oxythiamin effects mimicking the non-coenzyme action of thiamin.

#### 3.5.5. Different Resistance of Glioblastoma T98G and U87 Cells to Inhibitors of Central Metabolism is Due to the Cell-Specific Metabolism

Despite the complications in interpretation of changes in cellular NAD(P)H:resazurin reduction rates considered above, the assay is nevertheless useful to characterize metabolic differences between cells. In particular, using the assay, we showed that the two glioblastoma cell lines, T98G and U87, demonstrated different responses and resistance to inhibitors of the central metabolism. These findings are in line with independent studies, which established that T98G and U87 cells differ in expression of master metabolic regulators. For instance, T98G cells do not express the wild-type p53 protein [53]. Therefore T98G cells are relatively resistant to p53-mediated apoptosis [54], compared to U87 cells, expressing non-mutated p53. Another important gene, shown to be differently expressed and/or regulated by its activators Akt and MAPK in T98G and U87 cell lines, is HIF1α [55,56]. In particular, HIF1α is involved in the pyruvate dehydrogenase-dependent switch to hypoxic conditions [57,58,59]. Regarding up-regulated oncogenes and down-regulated tumor suppressors, U87 cell line has an expression pattern which is expected more for cancer cells, than that of T98G cells [60,61]. The data suggest that T98G cells have different mechanisms of oncotransformation, in agreement with their EGFR-independent fast growth [62] and resistance to known cancer therapies [63,64]. Our studies are in good accordance with these findings, as we show that, compared to U87 cells, T98G cells exhibit more metabolic similarity to normal cells. In particular, compared to U87 cells, T98G cells are less resistant to the inhibitors of the ThDP-dependent dehydrogenases (Figure 5) which are usually not considered to be active in cancer cells [52]. T98G cells are also more responsive to oxythiamin, as assessed by both the NAD(P)H:resazurin oxidoreductase (Figure 1) and enzymatic activities (Figure 2 and Figure 3). Furthermore, T98G cells do not express the high levels of OGDH during their growth, as U87 do (Table 1). However, unlike U87 cells, T98G cells up-regulate OGDH upon its inhibition (Figure 2). According to our previous studies [22,65], this feature is inherent in normal metabolism. When mammalian OGDH is inhibited *in vivo* to a critical level, which depends on the original, metabolism-specific OGDH expression, the enzyme is up-regulated to compensate for the inhibition. The data obtained in the current work on the glioblastoma cells, provide further examples of such up-regulation, dependent on both the inhibition and initial expression of OGDH. The up-regulation of OGDH by oxythiamin is more expressed in T98G cells (Figure 2) where the OGDH activity is lower, compared to U87 cells (Table 1). As a result, genetic background of the cell-specific metabolic and signaling pathways appears to contribute to the different cellular sensitivity to the inhibitors of central metabolism used in this work.
